# Coupling of the finite element limit equilibrium method with the stochastic search algorithm and its application for an embankment dam

**DOI:** 10.1038/s41598-026-45649-0

**Published:** 2026-05-09

**Authors:** Juraj Ŝtetiar, Juraj Chalmovský

**Affiliations:** Institute of Geotechnics, Faculty of Civil Engineering, Brno University of Technology, Veveří 331/95, 602 00 Brno, Czech Republic

**Keywords:** Finite element limit equilibrium method, Particle swarm optimization, Non-associated plasticity, Strength reduction technique, Slope stability analyses, Engineering, Mathematics and computing, Natural hazards

## Abstract

Slope stability analysis is a crucial task in the design of geotechnical structures. In many computational methods, such as those combining displacement-based finite element methods and strength reduction techniques, no assumptions are made a priori about the shape or position of the failure mechanism. This is usually considered to be a positive feature but there are situations where the ability to control the search domain for the critical slip surface might be of interest. These are the cases where the critical slip surface converges towards a local (unintended) one, or when it is necessary to determine the factor of safety for slopes with varying inclinations such as the upstream and downstream slopes of embankment dams. This article presents the Finite Element Limit Equilibrium Method (FELEM), which integrates stress states computed by the finite element method along a predefined trial slip surface to determine the factor of safety. This method is combined with a swarm-based metaheuristic optimization algorithm to identify the critical slip surface with the lowest factor of safety. The combined approach is tested on two numerical examples, addressing issues such as discretization error, optimization procedure settings, and non-associated plastic flow. Finally, the combination of FELEM and the optimization algorithm is applied to a boundary value problem of a newly designed embankment dam. Slope stability is evaluated independently for both the upstream and downstream slope, each with a different inclination. Computations are performed for the hydrostatic and steady-state seepage conditions.

## Introduction

Slope stability analysis is a fundamental task in the geotechnical engineering and it represents a significant portion of the calculations involved in the design of geotechnical structures. Numerous methods exist for calculating slope stability, each based on different physical principles, and their suitability can vary depending on the specific practical application.

The Limit Equilibrium Method (LEM), also known as the method of slices (Bishop ^1^), involves dividing the soil mass above a potential slip surface into multiple slices (Taylor ^2^). This division introduces a statically indeterminate problem, requiring assumptions regarding the forces acting between the slices (Krahn ^3^).

Although LEM is based on static analysis and does not account for displacements, several well-known approaches, including those by Bishop ^1^, Janbu ^4^, and Petterson ^5^, do not fully achieve both force and moment equilibrium (Duncan ^6^). Despite this limitation, LEM-based methods remain the most widely used for slope stability calculations. Methods that achieve complete equilibrium, such as those introduced by Morgenstern and Price ^7^, Spencer ^8^, Janbu ^9^, and Sarma ^10^, are considered rigorous (Duncan ^6^). The factor of safety (FoS) is typically calculated as the ratio of the resisting to driving forces along a specified slip surface. Because this calculation is related to a specific slip surface, iterative (Chen ^11^; Zhou and Cheng ^12^) or reliability methods (Xie ^13^; Hu et al. ^14^) are often employed to find the critical factor of safety.

The Strength Reduction Method (SRM) is a widely used technique in slope stability analysis, particularly when combined with numerical methods like the Finite Element Method (FEM) (Dawson ^15^; Griffiths and Lane ^16^) or the Discrete Element Method (DEM) (Huang et al. ^17^). SRM operates by incrementally reducing the soil’s strength until the equilibrium between internal and external forces is not fulfilled, with the factor of safety (FoS) calculated as the ratio of the initial to the most reduced strength parameters at equilibrium (Zienkiewicz ^18^; Matsui and San ^19^). Unlike Limit Equilibrium Methods, SRM does not require any assumptions about the position or shape of the slip surface, allowing it to identify the most critical slip surface (Griffiths and Marquez ^20^; Nian ^21^; Hu et al. ^22^).

**Fig. 1:**
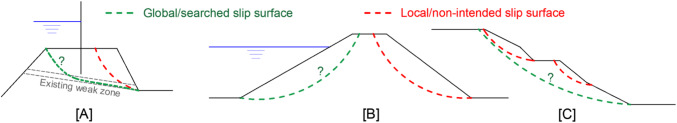
Motivation – The limitations of FE-SRM lie in its ability to calculate stability exclusively for the critical slip surface when solving slope stability problems.

However, a notable limitation of the FE-SRM combination is that it only identifies a single critical slip surface and its corresponding safety factor ($\textrm{FoS}_{\textrm{crit}}$). This can be restrictive in more complex scenarios, as illustrated in Fig. 1, where the presence of multiple potential failure surfaces requires their separate evaluation. Moreover, SRM primarily applies to the Mohr–Coulomb (MC) failure criterion, though it has been extended to more advanced constitutive models (Schneider-Muntau ^23^; Kadlíček and Maŝín ^24^). The method also faces challenges with the flow rule; while the associated flow rule may lead to a non-conservative FoS estimate, the more accurate non-associated flow rule can cause numerical instability, which can be mitigated using the Davis approach (Chen ^11^, Davis ^25^). Despite these challenges, SRM’s ability to simulate progressive failure and its integration with advanced modelling techniques make it a powerful tool, though further research is needed to address its limitations.

This paper presents an application of the FELEM (Finite Element Limit Equilibrium Method – Liu ^26^, Li ^27^, Fredlund ^28^, Krahn ^3^, Chugh ^29^, Stianson ^30^), which utilizes stresses calculated by the finite element method to calculate the factor of safety on a predefined slip surface. The position and shape of the trial slip surfaces are then continuously modified using mathematical optimization until the critical slip surface with the lowest value of FoS is found.

The FELEM algorithm, programmed in Python, is integrated with a stochastic optimization procedure known as Particle Swarm Optimization (PSO). PSO is a metaheuristic optimization technique responsible for updating the shape and position of trial slip surfaces within a predefined search region to identify the most critical one. The integration of PSO with Finite Element Limit Equilibrium Methods (FELEM) offers a robust and flexible approach for the identification of critical slip surfaces. PSO is a population-based stochastic optimization method inspired by the social behaviour of birds flocking or fish schooling. It optimizes a problem by iteratively refining candidate solutions based on a given measure of quality (Eberhart and Kennedy ^31^; Shi and Eberhart ^32^).

In the context of slope stability, PSO dynamically updates the position and shape of trial slip surfaces within a predefined search space. This search space is determined based on geological, geometrical, and material properties of the slope (Sakurai et al. ^33^). Unlike traditional deterministic methods, PSO enables the exploration of a larger, more complex search space, thereby increasing the probability of identifying the most critical slip surface – one that might be missed by conventional approaches (Cheng et al. ^34^).

PSO’s ability to handle nonlinear and discontinuous functions makes it particularly well-suited for slope stability problems where the failure mechanism is complex and difficult to predict (Jia et al. ^35^). The iterative nature of PSO, where each particle (potential solution) updates its position based on its own experience and that of its neighbours, allows for the global search of the critical slip surface with a reduced risk of converging on local minima (Li et al. ^36^).

However, the effectiveness of PSO in this application depends on the choice of its parameters, such as the number of particles, cognitive and social coefficients, and inertia weight, all of which directly influence the convergence speed and accuracy of the solution (Clerc ^37^; Eberhart and Shi ^38^). Additionally, other optimization techniques, such as Genetic Algorithms (GA) or Simulated Annealing (SA), and their hybridization, has been explored to enhance its performance, particularly in avoiding premature convergence and improving the precision of the identified critical slip surface (Shi et al. ^39^; Liu et al. ^40^, Zhang ^41^).

The basic theoretical aspects, including the generation of a trial slip surface, integration of shear and normal stresses for FoS evaluation, transformation of a non-associated problem into an associated one, and the PSO principle, are outlined first. The FELEM algorithm is then tested on numerical examples of homogeneous and heterogeneous slopes. In the case of the homogeneous slope, scenarios with both marginal and sufficient stability reserves are analyzed. Particular attention is paid to the influence of non-associated plasticity, mesh discretization error and PSO settings. These factors have not been or rarely analyzed in relation to FELEM-PSO. FELEM-PSO results are compared with calculations performed by FE-SRM and LEM. Finally, the FELEM-PSO algorithm is used for a stability assessment of a newly designed dam in the Czech Republic. FoS values are evaluated separately for the upstream and downstream slopes, which is not possible using the SRM method. In addition to the loading stages with an empty dam, the loading stage with a full reservoir and steady-state seepage conditions is also analyzed. Wherever possible, the critical slip surfaces and corresponding $\textrm{FoS}_{\textrm{crit}}$ values are compared with those obtained by SRM and LEM. Unless otherwise stated, all strength parameters are treated as effective.

**Fig. 2:**
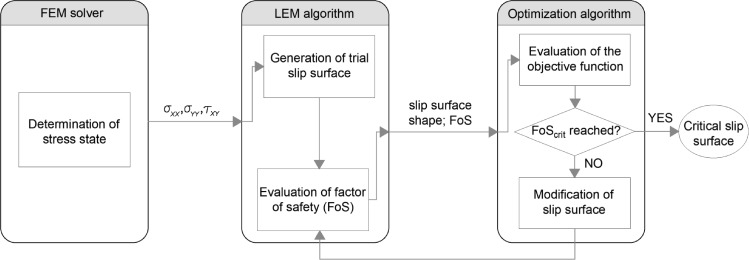
Flowchart of the FELEM-PSO method.

**Fig. 3:**
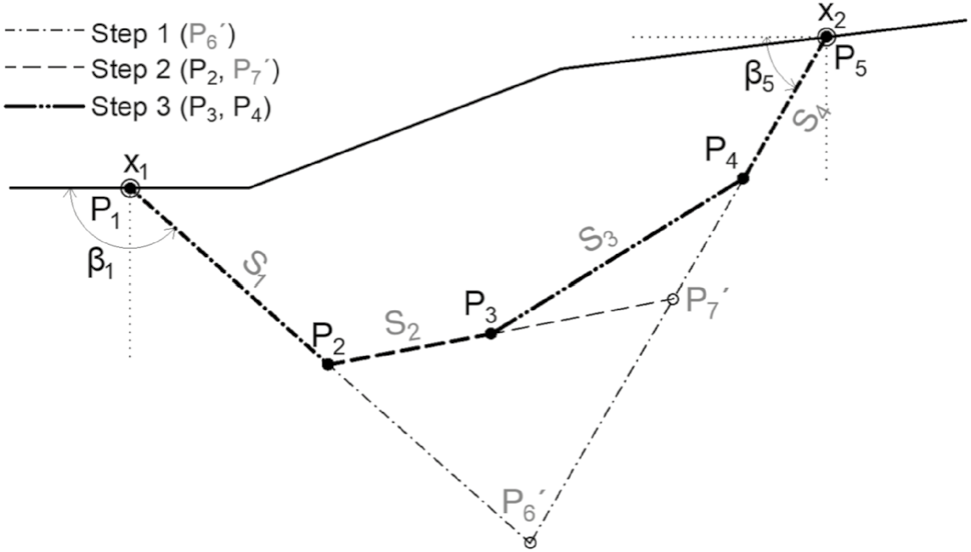
Adopted trial slip surface.

**Fig. 4:**
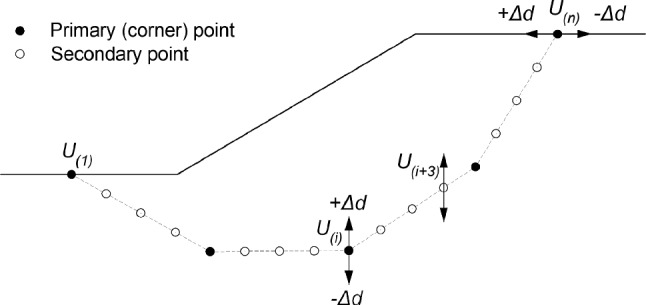
Primary and secondary governing points.

**Fig. 5:**
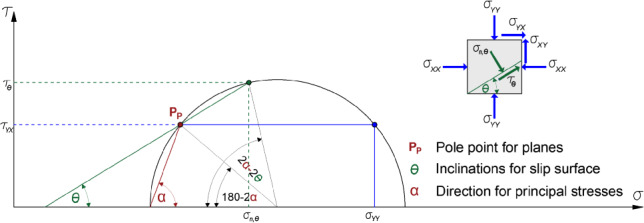
Transformation of Cartesian effective stresses.

**Fig. 6:**
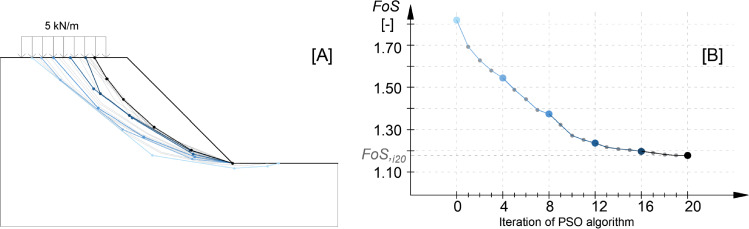
Variation of slip surface shape (**a**) and FoS (**b**) during the optimization process to find $\textrm{FoS}_{\text {crit}}$.

**Fig. 7:**
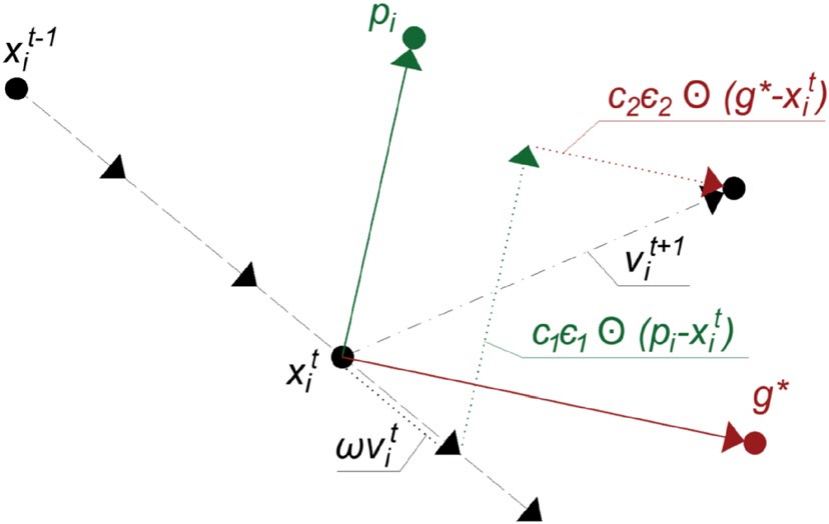
Particle velocity update based on previous velocity, local best position and global best position.

**Fig. 8:**
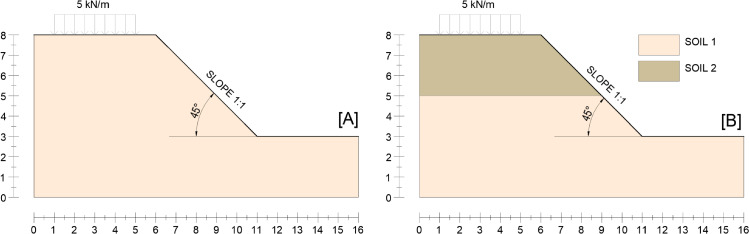
Geometry of two analysed examples: homogenous and heterogenous (layered) slope.

**Fig. 9:**
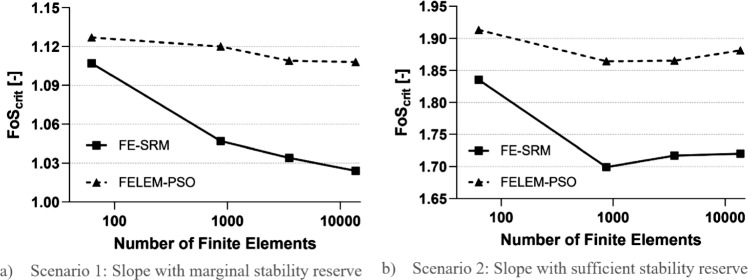
Influence of mesh discretization – homogenous slope.

## Algorithmization of the finite element limit equilibrium method

The finite element limit equilibrium method (FELEM) consists of three units: a) finite element solver, b) limit equilibrium (LEM) algorithm and c) optimization algorithm. The stress state for a given construction stage is computed using a finite element solver (Plaxis 2D 2023 was used in this study). The computed stress field is then exported to the LEM algorithm, which performs the following tasks: generation of a trial slip surface, iterative modification of the slip surface, and evaluation of the factor of safety. The LEM algorithm was programmed by the authors in Python programming language. The source code of the algorithm is available at https://doi.org/10.5281/zenodo.18674704. The results from the LEM algorithm are then passed to the optimization procedure, which adjusts the position and shape of the slip surface to minimize the objective function (i.e., to reach the lowest factor of safety, $\textrm{FoS}_{\textrm{crit}}$). For this purpose, the Particle Swarm Optimization (PSO) method ^42^ is used. The interaction of these three units is schematically shown in Fig. 2.

### Trial slip surface generation

The algorithm used for generation of trial slips surfaces is based on formulations presented by Li ^36^, Greco ^43^, Malkawi ^44^, Cheng ^34^. Each trial slip surface consists of four linear segments (S1 – S4) and thus five corner points (P1 – P5) as shown in Fig. 3. The geometry of the slip surface is completely defined by 8 governing parameters:

1 $$\begin{aligned} \boldsymbol{V} = (x_1, x_5, \beta _1, \beta _5, d_5, d_6, d_7, d_8) \end{aligned}$$

where $x_1$ and $x_5$ are longitudinal coordinates of the outer corner points P1 and P5, respectively. These are problem-dependent variables. Their values were chosen in such a way that they did not influence the optimization process (i.e., the critical slip surface did not converge towards these boundaries). The parameters $\beta _1$ and $\beta _5$ represent the inclinations of the two outer segments S1 and S4, respectively. The inclination $\beta _1$ is defined in the range $(\pi /2, \pi )$, while $\beta _5$ lies within the range $(0, \pi /2)$. The only constraint condition is that the intersection of the segments S1 and S4 must lie between points P1 and P5. The parameters $d_5$ to $d_8$ are random numbers in the range $(-0.5, 0.5)$, which guarantees that the individual segments of the trial slip surface will not cross each other. The variables $\beta _1$, $\beta _5$, $d_5$, $d_6$ and $d_8$ are generated randomly within the defined limits in the first iteration. Subsequently, their modification is controlled by the optimization algorithm. $\beta _1$ and $\beta _5$ are inclinations of outer segments S1 and S4, respectively and $d_5$ to $d_8$ are random numbers in range between $-0.5$ and 0.5.

A trial slip surface generation is divided into three steps (Fig. 3):

- **Step 1:** The position of a temporary governing point P6$'$ is determined based on the given parameters $x_1$, $x_5$, $\beta _1$ and $\beta _5$.
- **Step 2:** The position of the final governing point P2 and an additional auxiliary point P7$'$ is determined using the following equations:
  2 $$\begin{aligned} x_2 = d_5(x_1 - x_{6'}) + 0.5(x_1 + x_{6'}), \quad y_2 = d_5(y_1 - y_{6'}) + 0.5(y_1 + y_{6'}) \end{aligned}$$
  3 $$\begin{aligned} x_{7'} = d_6(x_5 - x_{6'}) + 0.5(x_5 + x_{6'}), \quad y_{7'} = d_6(y_5 - y_{6'}) + 0.5(y_5 + y_{6'}) \end{aligned}$$
- **Step 3:** Similar equations are used for determination of two final corner points P3, P4:
  4 $$\begin{aligned} x_3 = d_7(x_2 - x_{7'}) + 0.5(x_2 + x_{7'}), \quad y_3 = d_7(y_2 - y_{7'}) + 0.5(y_2 + y_{7'}) \end{aligned}$$
  5 $$\begin{aligned} x_4 = d_8(x_5 - x_{7'}) + 0.5(x_5 + x_{7'}), \quad y_4 = d_8(y_5 - y_{7'}) + 0.5(y_5 + y_{7'}) \end{aligned}$$

The positions of the primary points are not fixed, their values change within user-defined boundaries, which are governed by the PSO optimization algorithm to achieve the lowest value of FoS. To enhance the accuracy of the calculations, the primary corner points P1 to P5 are supplemented by secondary points (Fig. 4) along linear segments of the trial slip surface. The total number of points used in the stability analysis includes both primary and secondary points; however, the number of governing parameters in Eq. (1) is only influenced by the number of primary points. The polygonal trial slip consisting of four segments was chosen due to its reasonably low number of governing parameters and, at the same time, flexibility of the shape of the shear surface. However, it must be noted that that that more complex slip surfaces might occur. The presence of one or multiple weak layers with reduced strength parameters might control the overall slope stability. The influence of multiple weak stratums on the global stability of an open pit mine was analysed, for example, by Cala and Filisiak ^45^. The authors found significant differences in the $\textrm{FoS}_{\textrm{crit}}$ values and critical slip surfaces using the limit equilibrium method and the shear strength reduction methods. The complexity of shear surfaces may also be due to the fact that in some cases one initial slip surface might initiate another. The multi-slip surface technique in combination with the strength reduction method and Mohr–Coulomb yield surface was introduced e.g. by Zhang ^46^.

### Evaluation of factor of safety

The FoS value in the LEM algorithm is evaluated by means of numerical integration of the acting shear stress ($\tau _m^i$) and available shear strength ($\tau _f^i$) on the *i*-th segment along the generated polygonal trial slip surface (Eq. 6). $\Delta L^i$ is the segment length, and *n* is the total number of segments. The Mohr-Coulomb failure criterion is used to determine $\tau _f^i$ (Eq. 7), where $\sigma _n^i$ is the effective normal stress acting on the *i*-th segment, $c^i$ and $\varphi ^i$ are the effective shear strength parameters valid for the *i*-th segment.

6 $$\begin{aligned} \textrm{FoS} = \frac{\int \tau _f \, dL}{\int \tau _m \, dL} \end{aligned}$$

7 $$\begin{aligned} \tau _f^i = c^i + \sigma _n^i \tan \varphi ^i \end{aligned}$$

Based on Fig. 5, $\tau _m^i$ and $\sigma _n^i$ might be determined from Eq. 8. In these basic equations, it is possible to eliminate the radius of the Mohr circle (*r*) and direction of principal stresses ($\theta$) by using two additional formulas (Eq.  9). Thus, only the variables entering the calculation of $\tau _m^i$, $\sigma _n^i$ for each segment of the trial slip surface are the Cartesian stresses ($\sigma _{xx}^i$, $\sigma _{yy}^i$, $\tau _{xy}^i$) imported from the FEM solver and the slip surface geometry ($\alpha ^i$).

8 $$\begin{aligned} \tau _m^i = r \sin (2\alpha ^i - 2\theta ^i), \quad \sigma _n^i = \frac{1}{2}(\sigma _{yy}^i + \sigma _{xx}^i) - r \cos (2\alpha ^i - 2\theta ^i) \end{aligned}$$

9 $$\begin{aligned} \sin 2\alpha ^i = \frac{\tau _{xy}^i}{r}, \quad \tan 2\alpha ^i = \frac{-2 \tau _{xy}^i}{\sigma _{yy}^i - \sigma _{xx}^i} \end{aligned}$$

For the integral calculation, numerical integration is performed on the given data set using the composite trapezoidal rule, which approximates the area under the curve with trapezoids formed from the values on the curve.

### Associated and non-associated plastic flow

Convergence problems and numerical oscillations might occur when using non-associated plastic flow in the finite element analysis. These problems become more pronounced as the degree of non-associativity (difference between the effective friction $\varphi$ and the dilatancy angle $\psi$) increases. This is typical for steep slopes, such as rock slopes, where the effective friction angles are usually greater than $40^\circ$–$45^\circ$ (especially for small normal stresses). Davis ^25^ found out that, in the case of non-associated plasticity, the stress ratio ($\tau _1/\sigma _1$) at the failure determined from the Mohr stress circle is different from the stress ratio ($\tau _2/\sigma _2$) on the line of zero extension determined from the Mohr strain circle. The lines of zero extension represent the slip lines in the velocity field, with zero strain increments perpendicular to these lines. He therefore proposed to use reduced values of strength parameters, denoted as $c^*$ and $\varphi ^*$, in combination with associated plasticity:

10 $$\begin{aligned} \beta = \frac{\cos \varphi \cos \psi }{1 - \sin \varphi \sin \psi } \end{aligned}$$

11 $$\begin{aligned} c^* = \beta c, \quad \tan \varphi ^* = \beta \tan \varphi \end{aligned}$$

where $\beta$ is the reduction factor of the original effective strength characteristics $\varphi$ and *c*. This procedure is further noted as the “Davis approach”. Several authors have tested it for combination of the finite element method and the strength reduction method ^11,47^. In this paper, the Davis approach is incorporated into the FELEM-PSO algorithm, and its influence on the computed FoS is analyzed and compared with other methods.

## Identifying the critical slip surface: optimization algorithm and coupling with LEM

### Basic principle of the optimization method

The principles explained in the previous chapter serve to generate a trial slip surface and calculate the factor of safety for this particular surface. However, to evaluate the slope stability, it is necessary to find the critical slip surface with the lowest value of FoS (further noted as $\textrm{FoS}_{\textrm{crit}}$). This presents an optimization task where FoS is the objective function. The role of an optimization algorithm is to minimize the FoS value by adjusting the shape and position of the slip surface (schematically shown in Fig. 6). Particle Swarm Optimization, further noted as PSO ^31,42^, was utilized for this purpose.

The PSO method belongs to a group of metaheuristic evolutionary optimization procedures and is inspired by a collective decentralized intelligence of flock of birds or fishes searching for food. Each particle represents a possible solution. In the current case, this solution is the shape of slip surface specified by eight geometric parameters. The position of the particle in the search space is given by the values of these geometric inputs. For each particle in the swarm, the best position $p_i$ from all previous iterations, corresponding to a slip surface with the lowest FoS value, is stored in a memory (further referred to as local best or l-best). Similarly, the particle position $g^*$ with the lowest FoS value form all particles and iterations is stored and continuously updated (further noted as global best or g-best). Each particle has its velocity, given by Eq. 12, where $v_i^{t+1}$ is the velocity of *i*-th particle at the iteration $t+1$, $v_i^t$ and $x_i^t$ are the velocity and position of the particle in the previous iteration, respectively. $\varepsilon _1$, $\varepsilon _2$ are vectors of random numbers and $c_1$, $c_2$ are acceleration constants. Thus, the updated particle velocity is a function of the previous velocity, l-best and g-best. This process is schematically shown in Fig. 7.

12 $$\begin{aligned} v_i^{t+1} = v_i^t + c_1 \varepsilon _1 \odot (p_i - x_i^t) + c_2 \varepsilon _2 \odot (g^* - x_i^t) \end{aligned}$$

13 $$\begin{aligned} x_i^{t+1} = x_i^t + v_i^{t+1} \end{aligned}$$

The iteration process terminates when one of the following three situations occurs: the maximum number of iterations is reached, the required value of the objective function is reached or the changes in the objective function in successive iterations are smaller than a predefined limit value.

### Optimization procedure settings

The GBEST ^42,48^ variant of the PSO algorithm was adopted. Engelbrecht ^49^ performed a complex study of two basic neighbourhood topologies, namely the global best (GBEST) and local best (LBEST) PSO, using 60 boundary-constrained minimisation problems. He found out that, in general, both algorithms performed very similarly. GBEST PSO performed slightly better than LBEST PSO with respect to the success rate and efficiency. GBEST PSO was also utilized in several geotechnical applications, including slope stability analyses and critical slip surface searches ^34,36,50,51^. The dimension of the search space is defined by the size of the vector *V* (Eq. 1). Each set of values of the vector *V* defines one trial slip surface and presents a point (particle) in the multi-dimensional search space. The upper and lower bounds of variables in *V* vector were set individually for each analyzed geometry to avoid significantly restricting the optimization process. The maximum velocity parameter limits the maximum speed of a particle in one iteration, preventing overshooting the optimal solution and ensuring controlled exploration. The interval was set ^52,53^ to $(-10, 10)$ for each variable in vector *V*, meaning that particles can change their velocity by a maximum of $-10$ or 10 units per iteration.

Other important control variables are $c_1$ and $c_2$ factors, which are the weights for the cognitive and social components of the particles. Both were set to 2 according to recommendations^42,53^. The inertia weight $\omega$ defines the extent to which the particle retains its direction and velocity from the previous iteration. $\omega = 0.5$ was adopted ^54^, meaning a particle in the next iteration will retain half of its velocity from the previous iteration. The inertia damping coefficient $\omega _{\text {damp}}$ gradually reduces the inertia weight $\omega$ during the optimization process. In the analysis presented in this paper, inertia damping was not considered ($\omega _{\text {damp}} = 1$).

The topology profile defines how particles are connected and share information with each other, impacting the convergence speed and efficiency of the algorithm. The “Updated Star topology” recommended by Li ^55^ was utilized. The number of particles ($n_p$) and the number of iterations ($n_{it}$) were subjects of parametric studies, as these are problem-dependent variables, and their choice significantly influences the outcome of the optimization.

## Numerical examples

The FELEM-PSO algorithm was first tested on two numerical examples: (a) homogeneous slope consisting of one soil layer and (b) heterogeneous slope consisting of two soil layers with a horizontal interface between them (Fig. 8).

In both cases, a uniform load with a magnitude of 5 kN/m/m and a length of 4 m was applied at the crest. The stress state entering the LEM algorithm was obtained by the finite-element solver Plaxis 2D employing plain strain conditions. Fifteen-noded triangular elements with 12 Gauss points and cubic interpolation of displacements over a finite element were used unless otherwise specified. Strength parameters for both soil types are summarized in Table 1.

The results were compared with routinely used limit equilibrium methods Sarma^10^, Spencer^8^, Janbu^4,56^, and Morgenstern-Price^7,57^ and the finite element – strength reduction method. Two cases (referred to as the test scenarios) were analyzed for the homogeneous slope: I) slope with marginal stability reserve, close to failure (using Soil 1), II) slope with sufficient stability reserve (using Soil 2). For the heterogeneous slope, both materials were used simultaneously without distinguishing between their stability reserves.

**Table 1:** Geotechnical parameters for both examples.

Soil type	$\varphi '$ [$^\circ$]	$c'$ [kPa]	$\gamma$ [kN/m$^3$]	*ν* [-]
Soil 1	25	5	17	0.37
Soil 2	36	9	17	0.29

### Homogeneous slope

#### Influence of mesh density

**Fig. 10:**
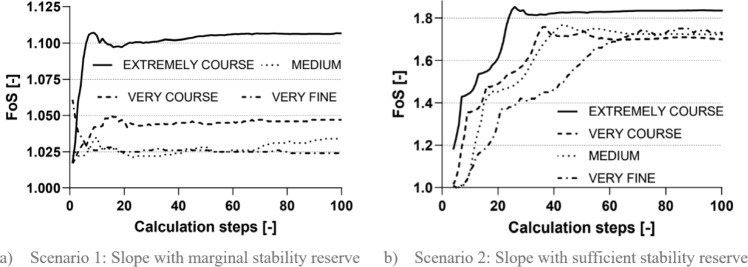
FoS development during strength reduction process (FE-SRM).

**Fig. 11:**
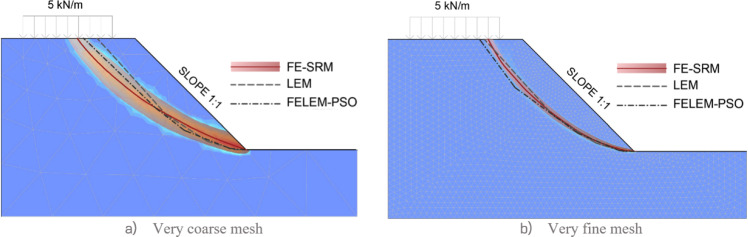
Comparison of critical slip surfaces obtained from FE-SRM, FELEM-PSO and LEM (Sarma) for homogenous slope.

**Fig. 12:**
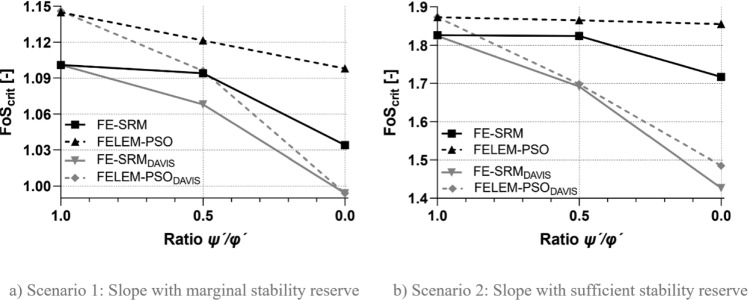
FoS from FE-SRM and FELEM-PSO for different $\psi /\varphi$ ratios – homogenous slope.

**Fig. 13:**
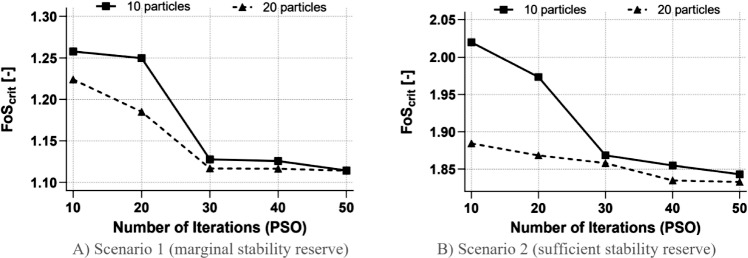
Comparison of FoS obtained with different number of particles and iterations.

**Fig. 14:**
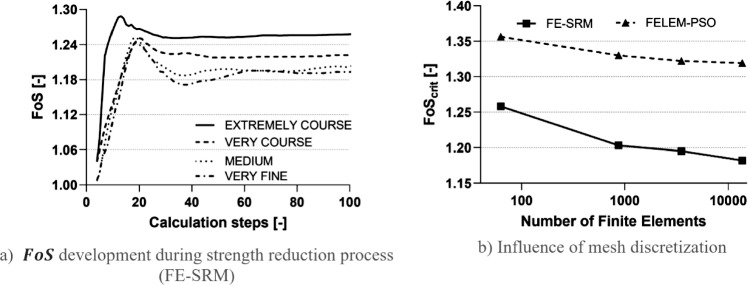
Influence of mesh discretization – heterogenous slope.

It is well known that too low mesh densities might result in overestimation of $\textrm{FoS}_{\textrm{crit}}$, particularly when finite elements with a lower degree of interpolation polynomial are used. In this section, the influence of mesh discretization is analyzed for four different mesh densities: extremely coarse (63 elements), very coarse (870 elements), medium (3514 elements) and very fine (13,675 elements). The results for both scenarios (with a marginal and sufficient stability reserve) are summarized in Table 2 and plotted in Fig. 9. The course of FoS over calculation steps is shown in Fig. 10, the calculation step states for the number of the consecutive incremental reduction of the shear strength parameters during the strength reduction process. The critical slip surfaces determined by both methods are compared in Fig. 11 for the coarsest and the finest finite element meshes. For comparison purposes, analyses for both test scenarios were performed also with four different limit equilibrium methods (Table 3). The critical slip surface from Sarma^10^ LEM is also plotted in Fig. 11.

For both test scenarios, the factor of safety decreases with increasing mesh density. It is interesting to note that for the slope with sufficient stability reserve, the factor of safety almost stabilizes, and only small changes are recorded between the coarse mesh and subsequent calculations with finer meshes. The FELEM-PSO method is less sensitive to the mesh density change compared to the FE-SRM. The difference between the very coarse and the very fine meshes is 1.7% and 8.1% (slope with marginal stability reserve), 2.6% and 6.7% (slope with sufficient stability reserve) for the FELEM-PSO and FE-SRM, respectively. This might be due to the fact that in the former case, a mesh discretization error is reflected only once during the stress state calculations by the finite element method. In the latter case, it is also involved in the strength reduction process, during which the finite element calculations are repeated for reduced strength parameters.

**Table 2:** FoS values evaluated for different mesh densities using FELEM-PSO and FE-SRM – homogenous slope.

Scenario	Method	Extremely coarse	Very coarse	Medium	Very fine
1	FE-SRM	1.107	1.047	1.034	1.024
2		1.835	1.699	1.717	1.720
1	FELEM-PSO	1.127	1.120	1.109	1.108
2		1.913	1.864	1.865	1.881

**Table 3:** FoS values for homogeneous slope determined by different types of LEM.

Scenario	Sarma	Spencer	Janbu	Morgenstern–Price
1	1.14	1.13	1.13	1.11
2	1.89	1.86	1.87	1.83

#### Influence of dilatancy angle and non-associated plasticity

To investigate the effect of non-associated plastic flow, three $\psi /\varphi$ ratios were used, namely 0, 0.5, and 1.0 (associated plasticity) for both scenarios. The results are summarized in Table 4 and in Fig. 12. The set of strength parameters denoted as $c^*, \varphi ^*, \psi = \varphi ^*$ means that, in all cases, the associated flow rule with the reduced strength parameters ($c^*, \varphi ^*$) according to the Davis approach^25^ is used. In the case of the FELEM-PSO approach, these reduced parameters were used both in the FE calculations of a stress state and in the subsequent but independent LEM algorithm. *c*, $\varphi$ and $\psi$ stand for the original strength parameters, with $\psi$ being the dilatancy angle based on the chosen $\psi /\varphi$ ratio.

When using the FELEM-PSO method, non-associated plasticity only affects the stress state calculated by the finite element method but does not influence the LEM algorithm, which uses the Mohr-Coulomb failure criterion with the original strength parameters *c*, $\varphi$. This contrasts with the FE-SRM, where non-associated plasticity affects both parts: the finite element calculation of the stress state and the subsequent strength reduction process, in which finite element calculations are performed again but with reduced strength parameters. $\textrm{FoS}_{\textrm{crit}}$ decreases with increasing $\psi /\varphi$ ratio in both test scenarios. Dilatancy cause positive volumetric changes (increase in volume), and if it is prevented (constrained dilatancy), e.g., by the soil above the slip surfaces, normal stresses and thus shear strength and factor of safety increases. The decrease in the factor of safety is more pronounced when the Davis approach, in combination with associated plasticity ($c^*, \varphi ^*, \psi =\varphi ^*$), is applied in both the FELEM-PSO and FE-SRM methods. Similar conclusions were reported by Tschuchnigg^47^ for the latter method. The reduced shear strength parameters $c^*$, $\varphi ^*$ directly influence the strength reduction process and the limit equilibrium algorithm in the case of the first and second method, respectively. The influence of the $\psi /\varphi$ ratio using the FELEM-PSO method and the non-associated plasticity ($c, \varphi , \psi$) is significantly smaller compared to those calculations with the Davis approach. This is because the non-associated plasticity only affects the stress state obtained by the finite element method, while in the LEM part of the algorithm non-reduced shear strength parameters (*c*, $\varphi$) are used.

**Table 4:** FoS values from FE-SRM and FELEM-PSO for different $\psi '/\varphi '$ ratios – homogeneous slope.

Method	Scenario	$\boldsymbol{\psi /\varphi }$		$\boldsymbol{\psi /\varphi }$	
		1	0.5	0	(c$^*$, $\varphi ^*$, $\psi = \varphi ^*$)
FE-SRM	1	1.101	1.068	1.034	0.994
	2	1.826	1.692	1.717	1.427
FELEM-PSO	1	1.145	1.096	1.098	0.994
	2	1.873	1.698	1.855	1.485

#### Influence of optimization procedure settings

Analyses were conducted for five different numbers of iterations $n_{\textrm{it}} = 10, 20, 30, 40, 50$ and two numbers of particles per iteration $n_{\textrm{p}} = 10, 20$. The obtained values of $\textrm{FoS}_{\textrm{crit}}$ are shown in Fig. 13 for both test scenarios. $\textrm{FoS}_{\textrm{crit}}$ decreases with increasing number of iterations. From a practical point of view, it is important to note that results are less sensitive to a number of iterations when a higher number of particles is used. Only small changes in $\textrm{FoS}_{\textrm{crit}}$ were detected when more than 30 iterations were considered. The number of iterations and particles should always be chosen individually and experimentally, based on the specific model or task. From the results, it can be deduced that in this case, a suitable ratio of $n_{\textrm{it}}/n_{\textrm{p}}$ lies between 1.5 to 2. Clerc^58^ proposed Eq. (14) for determining the minimum number of particles regardless the number of iterations, where $D_s$ is the dimension of the search space and [$\cdot$] is the integer calculator.

14 $$\begin{aligned} {n_{p,\min } = 10 + \left[ 2 \sqrt{D_s} \right] } \end{aligned}$$

Since $D_s = 8$ in the current case, the minimum recommended swarm size is $n_{p,\min } = 15$. Kalatehjari et al. ^50^ evaluated different PSO settings for a 3D slope stability problem with two geological units. Good convergence rate was obtained using 5, 15, 25, 35 and 55 particles. The best convergence and the highest average fitness were achieved with 35 particles after 65–70 iterations corresponding to $n_{it}/n_p = 1.86$–2.0. Chen et al. ^51^ found out that the convergence rate depends on the portion of the particle velocity from the previous iteration $v_i^t$ used to calculate the particle velocity in the next iteration $v_i^{t+1}$. If the entire value of $v_i^t$ was considered, FoS converged to its minimal value after 25 iterations using 10 particles ($n_{it}/n_p = 2.5$).

### Heterogeneous slope

Influence of the mesh discretization error, non-associated plasticity and optimization procedure in the FELEM-PSO method is further analyzed for a more realistic example of the heterogeneous slope with a horizontal boundary between two soil types. No specific test scenario is distinguished in this case. The model geometry and input parameters are specified in Chapter 3.

#### Influence of mesh density

**Fig. 15:**
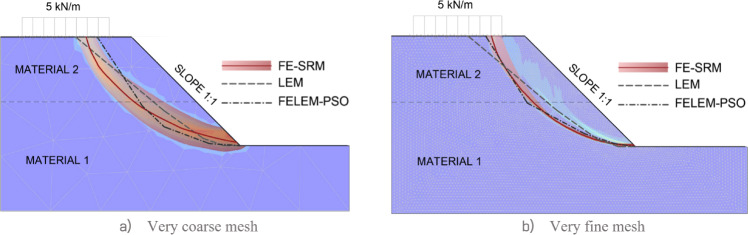
Comparison of critical slip surfaces obtained from FE-SRM, FELEM-PSO and LEM (Sarma) for heterogeneous slope.

The results of the mesh sensitivity analysis are summarized in Table 5 and in Fig. 14. The factors of safety obtained using various limit equilibrium methods are stated in Table 6. As with the homogeneous slope example, $\textrm{FoS}_{\textrm{crit}}$ decreases with the increasing mesh density in both methods. The total change between the extremely coarse and very fine mesh is 5.8% and 2.8% for FE-SRM and FELEM-PSO, respectively. In all cases, the latter method predicts $\textrm{FoS}_{\textrm{crit}}$ up to 10% higher compared to the former one. FELEM-PSO results are closer to the performed limit equilibrium analysis. The critical slip surfaces for very coarse and very fine mesh are compared in Fig. 15.

**Fig. 16:**
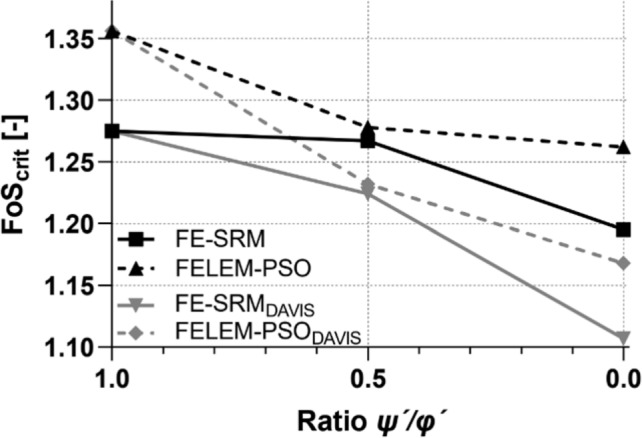
$\textrm{FoS}_{\text {crit}}$ from FE-SRM and FELEM-PSO for different $\psi '/\varphi '$ ratios – heterogenous slope.

**Fig. 17:**
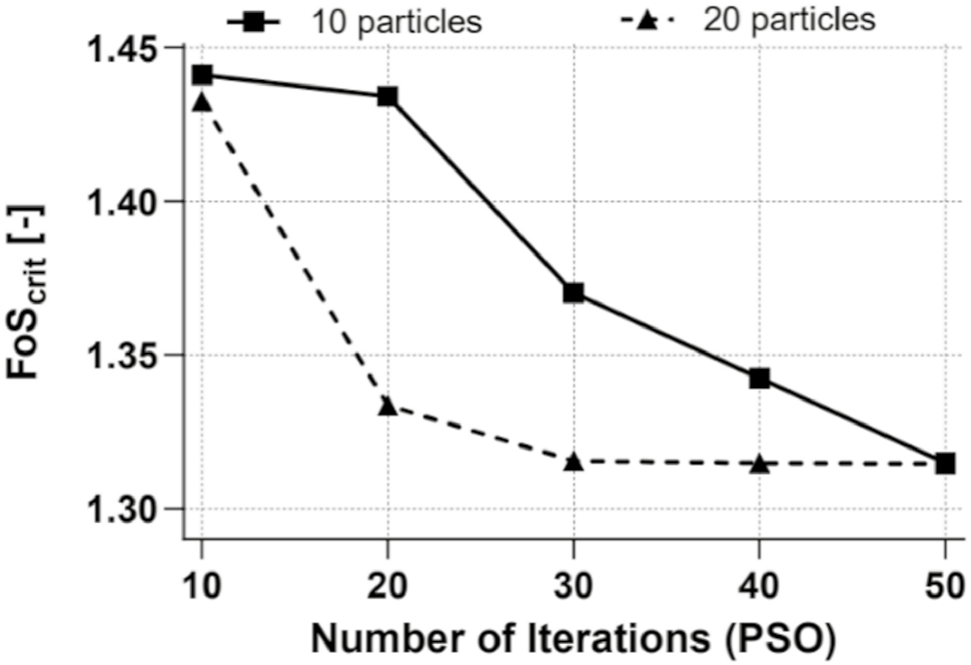
Comparison of FoS obtained with different settings for the number of particles and iterations (heterogeneous slope).

**Fig. 18:**
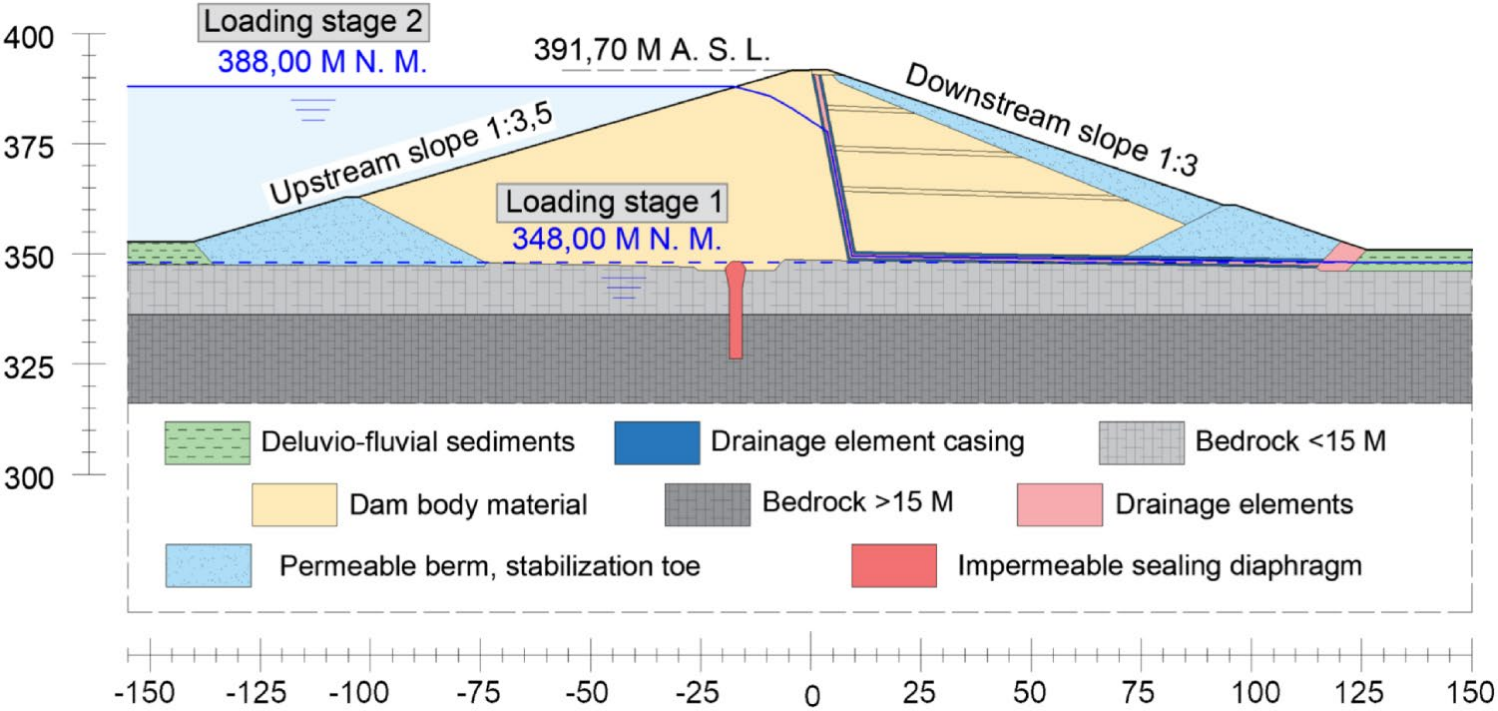
Cross section, construction materials, subsoil and water level for empty and full state.

**Fig. 19:**
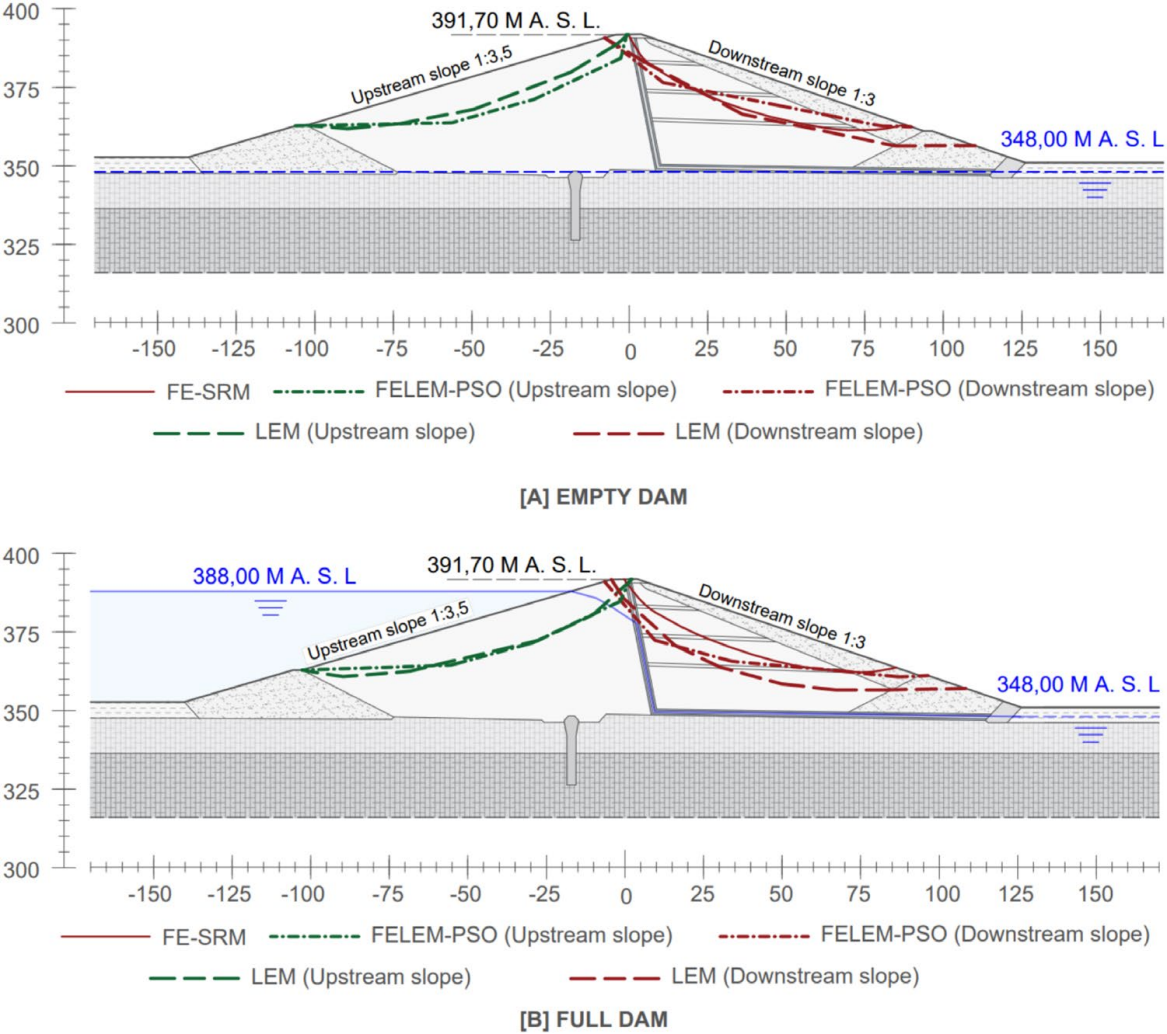
Critical slip surfaces determined by FELEM-PSO, FE-SRM and LEM.

**Fig. 20:**
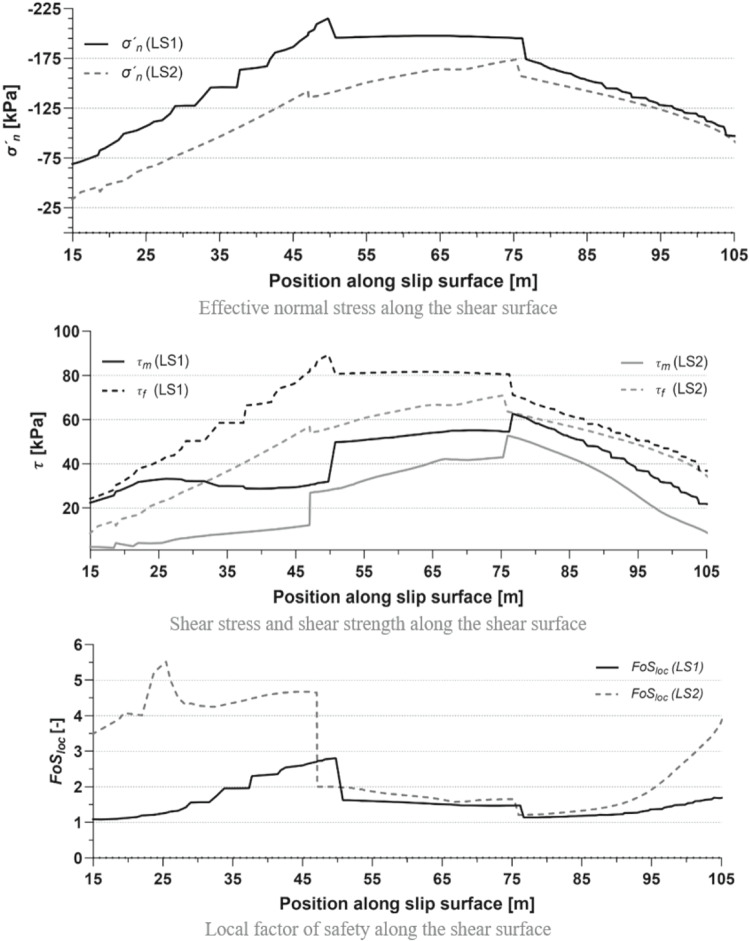
Stress state along critical slip surfaces.

**Table 5:** FoS values evaluated for different mesh densities using FELEM-PSO and FE-SRM – heterogeneous slope.

Scenario	Method	Extremely coarse	Very coarse	Medium	Very fine
2	FE-SRM	1.258	1.203	1.195	1.189
2	FELEM-PSO	1.356	1.330	1.322	1.319

**Table 6:** FoS values for heterogeneous slope determined by different types of LEM.

LEM	Sarma	Spencer	Janbu	Morgenstern–Price
FoS	1.36	1.32	1.30	1.30

#### Influence of dilatancy angle and non-associated plasticity

The calculations using FE-SRM and FELEM-PSO were repeated for three dilatancy angle to friction angle ratios $\psi /\varphi = 0, 0.5, 1.0$. Results are shown in Fig. 16 and further summarized in Table 7. The designation of individual calculations is the same as in Section 4.1. The results for the heterogeneous slope confirm the trends observed in the previous study on the homogeneous slope, where $\textrm{FoS}_{\textrm{crit}}$ decreases with decreasing $\psi /\varphi$ ratio. The lowest values of $\textrm{FoS}_{\textrm{crit}}$ were achieved using the FE-SRM method and associated plasticity with reduced strength parameters $c^*$, $\varphi ^*$ according to Davis^25^. Influence the non-associated plastic flow rule is smaller for FELEM-PSO compared to FE-SRM, as this factor is only considered during the finite element calculation of the stress field. On the contrary, the non-associated plastic flow affects both parts of the FE-SRM method – the finite element computation of the initial stress state and the shear strength reduction process.

**Table 7:** $\textrm{FoS}_{\textrm{crit}}$ from FE-SRM and FELEM–PSO for different $\psi /\varphi$ ratios – heterogeneous slope.

Method	$\boldsymbol{\psi /\varphi = 1}$		$\boldsymbol{\psi /\varphi = 0.5}$		$\boldsymbol{\psi /\varphi = 0}$
	$c, \varphi , \psi$	$c^*, \varphi ^*, \psi = \varphi ^*$	$c, \varphi , \psi$	$c^*, \varphi ^*, \psi = \varphi ^*$	$c^*, \varphi ^*, \psi = 0$
FE-SRM	1.275	1.267	1.224	1.195	1.107
FELEM-PSO	1.356	1.278	1.232	1.262	1.168

#### Influence of optimization procedure settings

As with the homogeneous slope, five levels of iteration counts and two levels of particle counts were used. As shown in Fig. 17, $\textrm{FoS}_{\textrm{crit}}$ values are more sensitive to a change in the number of iterations when using fewer particles. In the case of 50 iterations, stability factors are almost identical for both numbers of particles. From a practical point of view, when using 20 particles, there is only minor change in $\textrm{FoS}_{\textrm{crit}}$ when the number of iterations exceeds 30. It must be noted that the computations using FELEM-PSO method took longer than those performed by FE-SRM due to its iterative nature. In both numerical examples, the computational times for the FE-SRM were slightly less than one minute, analysis using FELEM-PSO typically took 3 to 4 minutes. Although these computations took longer time, they are still feasible form a practical point of view. Duration of calculations can be reduced by a) parallelization of computations and b) improvements in the critical slip search techniques. These two aspects are the subject of future development.

## Case study—stability of newly designed earth dam

The advantages of the FELEM-PSO method come into an importance especially in water structures such as embankment dams with different inclinations of the upstream and downstream slopes. In many cases, it is required to determine the factor of safety separately for both slopes. As previously discussed in the paper, this is not possible using the FE-SRM method without substantial modification of the finite element model. While it is partially feasible using LEM methods by restricting the geometry of the trial slip surfaces, the disadvantages of LEM methods remain. These include failure to satisfy all equilibrium conditions, limited modelling of construction stages, challenges in accounting for inhomogeneity, anisotropy and groundwater flow. On the contrary, the stress field entering the FELEM-PSO algorithm is obtained from the finite element method. It is possible to use advanced material models in the elastoplastic stress analysis, to consider soil suction in the unsaturated zone and stress changes during the water drawdown in the reservoir. In the LEM part of the process, the search area for slip surfaces might be restricted by specifying the coordinates ranges of their first and last points. In this way, the stability of both slopes of the dam can be addressed.

In the following section, the FELEM-PSO algorithm is utilized for the embankment dam Vlachovice ^59,60^ which is a newly designed dam in the south-east part of the Czech Republic.

### Project overview

The maximum height and width of the Vlachovice dam are 40 m and 266.5 m, respectively. The designed inclinations of the downstream and upstream slope are 1:3 and 1:3.5, respectively. Seepage through the embankment dam is controlled by the chimney and horizontal filter drains. Additional internal sand drains are situated on the downstream slope. The grout curtain is designed beneath the embankment body to prevent excessive seepage through the subsoil. The rockfill toes are designed on both sides of dam. The maximum water level ($H_{\textrm{max}}$) is 2 m below the level of the dam crest. Fig. 18 illustrates a schematic cross-section at the point of maximum dam height.

### Subsoil conditions and utilized constitutive models

The Elastoplastic Hardening Soil Model ^61,62^ with double shear and volumetric hardening was used for subsoils and construction materials of dam itself. Linear elastic – perfectly plastic Mohr-Coulomb model was adopted for highly fractured bedrock up to a depth of 15 m. Linear elasticity was assumed for depths greater than 15 meters. Unit weights, strength and hydraulic parameters are stated in Table 8, stiffness parameters are summarized in Table 9. The groundwater flow in the unsaturated zone is defined by a three-parameter Van Genuchten model ^63^, which is characterized by the residual saturation $S_{\textrm{res}}$, soil saturation below the groundwater level $S_{\textrm{sat}}$ ($S_{\textrm{sat}} = 1$ in the current case), and three fitting constants $g_a$, $g_n$, $g_c$. The residual saturation $S_{\textrm{res}}$ is considered as the ratio between the residual ($\theta _r$) and saturated ($\theta _s$) volumetric water content. Water transport through the unsaturated zone occurs mainly in the dam body (ID = 1), which is defined by the following parameters: $S_{\textrm{res}} = 0.0233$, $g_n = 1.254$, $g_a = 0.83$, $g_c = -0.5884$. Dam functional parts 2 to 4 are constructed from coarse-grained material and are defined by: $S_{\textrm{res}} = 0.062$, $g_n = 1.377$, $g_a = 3.83$, $g_c = 1.25$.

**Table 8:** Values of input parameters – unit weight, shear strength, hydraulic conductivity.

ID*	Dam functional part	$\gamma _{\textrm{unsat}}$ [kN/m$^3$]	$\gamma _{\textrm{sat}}$ [kN/m$^3$]	$c'$ [kPa]	$\varphi '$ [$^\circ$]	$k_x$ [m/s]	$k_y$ [m/s]
1	Dam body	16.8	20	6.4	24	$5.0\times 10^{-7}$	$1.0\times 10^{-7}$
2	Drainage elements	17	20	1	40	$1.0\times 10^{-3}$	$1.0\times 10^{-3}$
3	Permeable berm	19	21	1	40	$1.0\times 10^{-3}$	$1.0\times 10^{-3}$
4	Stabilizing toe	19	21	1	40	$1.0\times 10^{-3}$	$1.0\times 10^{-3}$
5	Impermeable diaphragm	25	25	–	–	$1.0\times 10^{-9}$	$1.0\times 10^{-9}$
A	Deluvio-fluvial sediments	17	20	5	24	$1.0\times 10^{-7}$	$1.0\times 10^{-7}$
B	Bedrock < 15 m	18	20	1	28	$1.0\times 10^{-7}$	$1.0\times 10^{-7}$
C	Bedrock > 15 m	18	20	–	–	$1.0\times 10^{-8}$	$1.0\times 10^{-8}$

*1 to 5 – construction layers, A to C – subsoil layers.

**Table 9:** Values of input parameters – deformation parameters.

ID*	Dam functional part	$E_{50}^{\textrm{ref}}$ [MPa]	$E_{\textrm{ur}}^{\textrm{ref}}$ [MPa]	*m* [-]	$\nu '/\nu _{\textrm{ur}}$ [-]
1	Dam body	10	30	1.0	0.2
2	Drainage elements	70	210	0.5	0.2
3	Permeable berm	50	150	0.5	0.2
4	Stabilizing toe	100	300	0.5	0.2
5	Impermeable diaphragm	50	–	–	0.2
A	Deluvio-fluvial sediments	20	60	0.5	0.2
B	Bedrock < 15 m	75	–	–	0.25
C	Bedrock > 15 m	75	–	–	0.25

*1 to 5 – construction layers, A to C – subsoil layers.

### Construction stages, adopted methods and analyzed scenarios

Two constructions stages were analyzed:

- *LS 1* – the dam construction is completed, and the primary consolidation has taken place. The ground water level is at the level before the dam was built (348 m a.s.l.) and hydrostatic conditions are considered.
- *LS 2* – the dam is full, with the water level in the reservoir at 388.0 m a.s.l. Steady-state conditions are considered, with no excess pore pressures due to undrained loading.

Apart from the FELEM-PSO method, the FE-SRM and LEM methods (Sarma) were utilized. The FELEM-PSO algorithm was used separately for the upstream and downstream slope in each analyzed case.

### Results and discussion

The computed $\textrm{FoS}_{\textrm{crit}}$ values are summarized in Table 10 and the positions of the critical slip surfaces are compared in Fig. 19 for both loading stages. In general, the differences between FE-SRM and FELEM-PSO are small. The $\textrm{FoS}_{\textrm{crit}}$ for the upstream slope is not evaluated in the case of FE-SRM as this method identifies only the critical slip surface which is in the steeper downstream slope. In the first loading stage, FELEM-PSO correctly predicts higher $\textrm{FoS}_{\textrm{crit}}$ for the upstream slope (1.818) compared to the steeper downstream slope (1.798).

An increase in the water level in the reservoir had a minimal effect on the downstream slope stability (reduction of $\textrm{FoS}_{\textrm{crit}}$ from 1.798 to 1.752), as the chimney drain effectively reduces pore pressures in this area. On the contrary, the increase in the water level led to the substantial $\textrm{FoS}_{\textrm{crit}}$ increase in the case of the upstream slope predicted by the FELEM-PSO (from 1.818 to 2.089). In the case of LEM, the increase was marginal (1.84 to 1.88).

To justify this result, the stress state along the slip surface was analyzed for both construction stages. Distributions of the effective normal stresses $\sigma '_n$, acting (mobilized) shear stress $\tau _m$, available shear strength $\tau _f$ and local safety factor $\textrm{FoS}_{\textrm{loc}} = \tau _f / \tau _m$ are shown in Fig. 20. Filling the reservoir led to a decrease in the effective stress and in the available shear strength in the upstream area due to water buoyancy. This effect alone would cause a drop in $\textrm{FoS}_{\textrm{crit}}$ between the loading stages 1 and 2. However, a more dominant effect is that the mobilized shear stress decreases along the critical slip surface when the reservoir is full. This is probably due to the stabilizing effect of the external water pressure. To a certain extent, the water in reservoir acts as the counter-load against the self-weight of the embankment dam. This resulted in an increase of $\textrm{FoS}_{\textrm{loc}}$ in the upstream area in the second loading stage.

**Table 10:** Comparison of obtained $\textrm{FoS}_{\textrm{crit}}$ values.

Stage	Slope	FE-SRM	FELEM-PSO	LEM (Sarma)
1 Empty dam after construction	Upstream	–	1.818	1.84
	Downstream	1.735–1.742	1.798	1.82
2 Storage water level, steady flow	Upstream	–	2.089	1.88
	Downstream	1.738–1.746	1.752	1.84

## Conclusions

In this study, the finite element limit equilibrium method (FELEM) was combined with the particle swarm optimization (PSO) procedure. The main advantage of FELEM is that it uses a stress state computed via the finite element method, which allows an application of advanced material models, construction staging, hydraulic coupling, etc., while still maintaining control over the position of trial slip surfaces. The following main conclusions are drawn:

- $\textrm{FoS}_{\textrm{crit}}$ decreases with increasing degree of non-associativity, however this decrease is smaller in FELEM–PSO compared to FE–SRM. The probable reason for this is that, in the FE–SRM method the non-associated plastic flow affects both the initial stress state calculation and the subsequent strength reduction process, whereas in the FELEM–PSO method, the LEM part of the algorithm is unaffected by the flow rule choice.
- Approximation of the non-associated plastic flow with the associated plasticity and reduced strength parameters according to Davis^25^ approach led to the lower values of $\textrm{FoS}_{\textrm{crit}}$ both in the FELEM–PSO and FE–SRM.
- Sufficiently accurate factors of safety were achieved after 30 iterations with 10 or 20 particles. The appropriate $n_{\textrm{it}}/n_{\textrm{p}}$ ratio was estimated to be between 1.5 and 2 based on this study.
- Application of the FELEM–PSO for a newly designed embankment dam made it possible to evaluate the factor of safety of the downstream and upstream slope independently. This is not possible by strength reduction techniques as these identify only the slip surface with the lowest value of FoS. The increase in water level led to an increase in stability of the upstream slope. Despite the reduction of effective normal stresses due to the water buoyancy and a corresponding decrease in the available shear strength along the slip surface, the acting shear stresses were also reduced due to the stabilizing effect of the external water pressure acting on the upstream slope.
- Further development of the FELEM–PSO should include parallelization of computations and utilization of newly emerging metaheuristic optimization methods to reduce computational time. The analyses presented in this study were treated as single objective optimization tasks. Multi-objective optimization procedures (MOOP) are of particular interest for newly built earth structures as they can incorporate multiple additional requirements in the optimality criteria such as the construction cost, CO$_2$ emissions etc.

## Data Availability

The source files of the finite element models used in the current study can be accessed using the following link: 10.5281/zenodo.19067463 (DOI: 10.5281/zenodo.19067463) or are available from the corresponding author Juraj Stetiar via e-mail 212375@vutbr.cz. The source code for the FELEM algorithm and corresponding scripts might be found in 10.5281/zenodo.18674703 (DOI: 10.5281/zenodo.18674703).

## Code availability

The source code for the FELEM algorithm and Python - Plaxis scripts of the boundary value problems has been uploaded to Zenodo and assigned a unique permanent DOI: 10.5281/zenodo.18674703 (access: 10.5281/zenodo.18674703).

## References

[1] Bishop, A. The use of the slip circle in the stability analysis of slopes. *Géotechnique* **5**, 7–17. 10.1680/geot.1955.5.1.7 (1954).

[2] Taylor, D. Stability of earth slopes. *J. Boston Soc. Civ. Eng.* **24**, 197–247 (1937).

[3] Krahn, J. The 2001 r.m. hardy lecture: The limits of limit equilibrium analyses. *Can. Geotech. J.* **40**, 643–660. 10.1139/t03-024 (2001).

[4] Janbu, N. Application of composite slip surface for stability analysis. In *Proc. European Conf. on Stability of Earth Slopes*, Vol. 3, 43–49 (1954).

[5] Petterson, K. The early history of circular sliding surfaces. *Géotechnique* **5**, 275–296. 10.1680/geot.1955.5.4.275 (1955).

[6] Duncan, J. State of the art: Limit equilibrium and finite-element analysis of slopes. *J. Geotech. Eng. ASCE* **122**, 577–596. 10.1061/(ASCE)0733-9410(1996)122:7(577) (1996).

[7] Morgenstern, N. & Price, V. The analysis of the stability of general slip surfaces. *Géotechnique* **15**, 79–93. 10.1680/geot.1965.15.1.79 (1965).

[8] Spencer, E. A method of analysis of the stability of embankments assuming parallel interslice forces. *Géotechnique* **17**, 11–26. 10.1680/geot.1967.17.1.11 (1967).

[9] Janbu, N. *Slope Stability Computations* 47–86 (Wiley, 1973).

[10] Sarma, S. Stability analysis of embankments and slopes. *Géotechnique* **23**, 423–433 (1973).

[11] Chen, X., Wang, D., Yu, Y. & Lyu, Y. A modified Davis approach for geotechnical stability analysis involving non-associated soil plasticity. *Geotechnique* **70**, 1109–1119. 10.1680/jgeot.18.p.158 (2020).

[12] Zhou, X. & Cheng, H. Analysis of stability of three-dimensional slopes using the rigorous limit equilibrium method. *Eng. Geol.* **160**, 21–33. 10.1016/j.enggeo.2013.03.027 (2013).

[13] Xie, M., Esaki, T. & Cai, M. Gis-based implementation of three-dimensional limit equilibrium approach of slope stability. *J. Geotech. Geoenviron. Eng.* **132**, 656–660. 10.1061/(ASCE)1090-0241(2006)132:5(656) (2006).

[14] Hu, Y. et al. First order reliability-based design optimization of 3d pile-reinforced slopes with pareto optimality. *Comput. Geotech.* **162**, 105635. 10.1016/j.compgeo.2023.105635 (2023).

[15] Dawson, E., Roth, W. & Drescher, A. Slope stability by strength reduction. *Géotechnique* **49**, 835–840. 10.1680/geot.1999.49.6.835 (1999).

[16] Griffiths, D. & Lane, P. Slope stability analysis by finite elements. *Géotechnique* **49**, 387–403. 10.1680/geot.1999.49.3.387 (1999).

[17] Huang, X., Wang, G. & Zhang, L. An analytical solution for critical sliding surface of stepped rock slope: A case study of Xinjing coal mine landslide. *Bull. Eng. Geol. Env.* **84**, 78. 10.1007/s10064-024-04079-w (2025).

[18] Zienkiewicz, C., Humpheson, C. & Lewis, R. Associated and non-associated visco-plasticity and plasticity in soil mechanics. *Tech. Rep.* **25**, 671–689 (1975).

[19] Matsui, T. & San, K. Finite element slope stability analysis by shear strength reduction technique. *Soils Found.* **32**, 59–70. 10.3208/sandf1972.32.59 (1992).

[20] Griffiths, D. & Marquez, R. Three-dimensional slope stability analysis by elasto-plastic finite elements. *Géotechnique* **57**, 537–546. 10.1680/geot.2007.57.6.537 (2007).

[21] Nian, T., Huang, R., Wan, S. & Chen, G. Three-dimensional strength reduction finite element analysis of slopes: Geometric effects. *Can. Geotech. J.* **49**, 574–588. 10.1139/t2012-014 (2012).

[22] Hu, Y., Sun, Z. & Ji, J. Pseudo-dynamic stability analysis of 3d rock slopes considering tensile strength-modified hoek-brown failure criterion: Seismic ubla implementations. *Eng. Geol.* **339**, 107786. 10.1016/j.enggeo.2024.107786 (2024).

[23] Schneider-Muntau, B., Medicus, G. & Fellin, W. Strength reduction method in barodesy. *Comput. Geotech.* **95**, 57–67. 10.1016/j.compgeo.2017.09.010 (2018).

[24] Kadlíček, T. & Mašín, D. The strength reduction method in clay hypoplasticity. *Comput. Geotech.*10.1016/j.compgeo.2020.103687 (2020).

[25] Davis, E. Theories of plasticity and failure of soil masses. In *Soil Mechanics: Selected Topics* (ed. Lee, I.) 341–354 (Elsevier, 1968).

[26] Liu, S., Shao, L. & Li, H. Slope stability analysis using the limit equilibrium method and two finite element methods. *Comput. Geotech.* **63**, 291–298 (2015).

[27] Li, H. *et al.* Particle swarm optimization algorithm coupled with finite element limit equilibrium method for geotechnical practices. *Math. Probl. Eng.* (2012).

[28] Fredlund, D. & Scoular, R. Using limit equilibrium concepts in finite element slope stability analysis. In *Int. Symp. on Slope Stability Engineering*, 31–47 (1999).

[29] Chugh, A. On the boundary conditions in slope stability analysis. *Int. J. Numer. Anal. Methods Geomech.* **27**, 905–926. 10.1002/nag.305 (2003).

[30] Stianson, J., Fredlund, D. & Chan, D. Three-dimensional slope stability based on stresses from a stress-deformation analysis. *Can. Geotech. J.* **48**, 891–904. 10.1139/t11-006 (2011).

[31] Eberhart, R. & Kennedy, J. A new optimizer using particle swarm theory. In *Proc. Sixth Int. Symp. on Micro Machine and Human Science*, 39–43 (1995).

[32] Shi, Y. & Eberhart, R. A modified particle swarm optimizer. In *Proc. 1998 IEEE Int. Conf. on Evolutionary Computation*, 69–73 (1998).

[33] Sakurai, T., Takewaki, I. & Yokoyama, H. A particle swarm optimization approach for optimal design of short-term seismic retrofit strategy of buildings. *Eng. Struct.* **25**, 1569–1577 (2003).

[34] Cheng, Y., Chi, S. & Li, L. Particle swarm optimization algorithm for the location of the critical non-circular failure surface in two-dimensional slope stability analysis. *Comput. Geotech.* **34**, 92–103 (2007).

[35] Jia, H., Li, D. & Zhang, L. A hybrid particle swarm optimization method for locating the critical slip surface in slope stability analysis. *Struct. Multidiscip. Optim.* **38**, 239–250 (2009).

[36] Li, D., Zhang, L. & Tang, X. A particle swarm optimization algorithm for locating the critical slip surface of slopes. *Can. Geotech. J.* **49**, 577–588 (2012).

[37] Clerc, M. *Particle Swarm Optimization* (Wiley, 2006).

[38] Eberhart, R. & Shi, Y. Particle swarm optimization: developments, applications and resources. In *Proc. 2001 Congress on Evolutionary Computation*, 81–86 (2001).

[39] Shi, Z., Chen, Q. & Tang, H. A hybrid optimization algorithm based on particle swarm optimization and genetic algorithm. *Comput. Geotech.* **57**, 59–66 (2014).

[40] Liu, H., Li, H., Zhang, C. & Li, D. A hybrid particle swarm optimization algorithm for slope stability analysis. *Eng. Geol.* **202**, 1–9 (2016).

[41] Zhang, Z. Optimization of the critical slip surface of three-dimensional slope by using an improved genetic algorithm. *Int. J. Geomech.* **20**, 04020120 (2020).

[42] Kennedy, J. & Eberhart, R. Particle swarm optimization. In *Proc. IEEE Int. Conf. on Neural Networks*, 1942–1948 (1995).

[43] Greco, V. Efficient Monte Carlo technique for locating critical slip surface. *J. Geotech. Eng.* **122**, 517–525 (1996).

[44] Malkawi, A., Hassan, W. & Sarma, S. Global search method for locating general slip surface using Monte Carlo techniques. *J. Geotech. Geoenviron. Eng.* **127**, 688–698 (2001).

[45] Cala, M. & Filisiak, J. Analysis of slope stability in open pit mines using the shear strength reduction technique. *Int. J. Rock Mech. Min. Sci.* **40**, 643–655. 10.1016/S1365-1609(03)00056-3 (2003).

[46] Zhang, Y., Li, X. & Wang, J. Multi-slip surfaces searching method for slope stability analysis considering weak interlayers. *Comput. Geotech.* **149**, 104812. 10.1016/j.compgeo.2022.104812 (2022).

[47] Tschuchnigg, F., Schweiger, H. & Sloan, S. Slope stability analysis by means of finite element limit analysis and finite element strength reduction techniques. Part I: Numerical studies considering non-associated plasticity. *Comput. Geotech.* **70**, 169–177. 10.1016/j.compgeo.2015.06.018 (2015).

[48] Eberhart, R. & Shi, Y. Comparing inertia weights and constriction factors in particle swarm optimization. In *Proc. 2000 Congress on Evolutionary Computation*, 84–88, 10.1109/CEC.2000.870279 (2000).

[49] Engelbrecht, A. P. Particle swarm optimization: Global best or local best? In *2013 BRICS Congress on Computational Intelligence, 11th Brazilian Congress on Computational Intelligence*, 124–135 (2013).

[50] Kalatehjari, R., Ali, N. & Kholghifard, M. The contribution of particle swarm optimization to three-dimensional slope stability analysis. *Comput. Geotech.* **61**, 38–52. 10.1016/j.compgeo.2014.05.005 (2014). 10.1155/2014/973093 PMC4058805 24991652

[51] Chen, W. W., Shen, Z.-P., Wang, J.-A. & Tsai, F. Scripting stabl with pso for analysis of slope stability. *Neurocomputing* **148**, 167–174. 10.1016/j.neucom.2012.10.048 (2015).

[52] Koudela, P. *Determination of Input Parameter Values for Advanced Material Models Using Optimization Methods (in Czech)*. Master’s thesis,Brno University of Technology, Faculty of Civil Engineering, Institute of Geotechnics (2018). Thesis Supervisor: Ing. Juraj Chalmovský, Ph.D.

[53] Bentsen, C. Package ‘pso’. https://cran.r-project.org/web/packages/pso/pso.pdf (2024). Accessed 30 Oct 2024.

[54] Bansal, J. *et al.* Inertia weight strategies in particle swarm optimization. In *Proceedings of the 2011 Third World Congress on Nature and Biologically Inspired Computing*, 19–21, 10.1109/NaBIC.2011.6089659 (2011).

[55] Li, X.-L., Serra, R. & Olivier, J. An investigation of particle swarm optimization topologies in structural damage detection. *Appl. Sci.* **11**, 5144. 10.3390/app11115144 (2021).

[56] Janbu, N. *Slope Stability Computations* (Wiley, 1973).

[57] Morgenstern, N. & Price, V. A numerical method for solving the equations of stability of general slip surfaces. *Comput. J.* **9**, 388–393 (1967).

[58] Clerc, M. Standard particle swarm optimisation. *Particle Swarm Central* (2012). Standard PSO definition.

[59] Aquatis, a.s. Vlára, vlachovice water structure. technical and economic study. Tech. Rep., Aquatis a.s., Brno (2015).

[60] Říha, J., Kotaška, S. & Mezera, J. Vlachovice water structure. technical assistance. possibilities of alternative material use for the dam. Tech. Rep., Brno (2020).

[61] Schanz, T. *Zur Modellierung des mechanischen Verhaltens von Reibungsmaterialien: eine erweiterte Hypoplastizitätstheorie*. Ph.D. thesis, Universität Karlsruhe (TH) (1998).

[62] Schanz, T., Vermeer, P. & Bonnier, P. The hardening soil model: Formulation and verification. *Proceedings of the International Symposium “Beyond 2000 in Computational Geotechnics”* 281–296 (1999).

[63] van Genuchten, M. A closed-form equation for predicting the hydraulic conductivity of unsaturated soils. *Soil Sci. Soc. Am. J.* **144**(5), 892–898 (1980).

